# Biological Characteristics and Functional Analysis of the Linoleic Acid Synthase Gene *ZjFAD2* in Jujube

**DOI:** 10.3390/ijms242015479

**Published:** 2023-10-23

**Authors:** Junjun Jiang, Qianqian Shi, Xi Li, Xueying He, Cuiyun Wu, Xingang Li

**Affiliations:** 1 College of Forestry, Northwest A&F University, Xianyang 712100, China; jiangjunjun@nwafu.edu.cn (J.J.);; 2 Research Center for Jujube Engineering and Technology National Forestry and Grassland Administration, Xianyang 712100, China; 3 Key Comprehensive Laboratory of Forestry of Shaanxi Province, Northwest A&F University, Xianyang 712100, China; 4 College of Horticulture and Forestry, Tarim University, Alar 843300, China

**Keywords:** jujube, fatty acids, linoleic acid synthesis, *FAD2* genes

## Abstract

Jujube fruit is rich in linoleic acid and other bioactive components and has great potential to be used for the development of functional foods. However, the roles of *FAD2* genes in linoleic acid biosynthesis in jujube fruit remain unclear. Here, we identified 15 major components in jujube and found that linoleic acid was the main unsaturated fatty acid; major differences in the content and distribution of linoleic acid in the pulp and seeds were observed, and levels of linoleic acid decreased during fruit maturation. Analysis of the fatty acid metabolome, genome, and gene expression patterns of cultivated and wild-type jujube revealed five *ZjFAD2* family members highly related to linoleic acid biosynthesis. The heterologous expression of these five *ZjFAD2* family members in tobacco revealed that all five of these genes increased the content of linoleic acid. Additionally, transient expression of these genes in jujube fruit and the virus-induced gene silencing (VIGS) test further confirmed the key roles of *ZjFAD2-11* and *ZjFAD2-1* in the biosynthesis of linoleic acid. The results of this research provide valuable insights into the molecular mechanism underlying linoleic acid synthesis in jujube and will aid the development of quality-oriented breeding strategies.

## 1. Introduction

Jujube (*Ziziphus jujuba* Mill.), which belongs to the *Rhamnaceae* family, is an economically important tree species. It is native to China and has been cultivated and utilized for over 7000 years [1,2]. Jujube fruits are rich in nutrients and bioactive compounds, including flavonoids, vitamin C, alkaloids, triterpenic acids, and fatty acids [3,4,5]. These natural components contribute to its various health benefits, including its antioxidant, anti-allergic, immune-enhancing, and vasodilatory effects, and these health-promoting properties have made jujube highly popular among consumers [6,7]. Linoleic acid is an essential nutrient known to play a key role in human health because it cannot be synthesized endogenously in the human body and needs to be obtained through dietary sources. Therefore, the presence of linoleic acid in jujube fruits adds to their nutritional value and health benefits.

Linoleic acid is an abundant polyunsaturated fatty acid in jujube fruits, especially concentrated in the seeds compared with the pulp [8]. It is an omega-6 fatty acid and a pivotal component of the nutritional profile of jujube fruits, as it has been shown to confer considerable human health benefits. Linoleic acid is a precursor to other important omega-3 fatty acids, such as eicosapentaenoic acid (EPA) and docosahexaenoic acid (DHA), and thus plays a key role in brain development, nervous system development, and vision. Moreover, linoleic acid serves as a precursor to the prostaglandin E series and plays a crucial role in inflammatory reactions. Adequate intake of linoleic acid may effectively regulate inflammatory responses [9,10] and provide cardiovascular benefits, including lowering blood pressure, improving blood lipid profiles, and reducing the risk of heart disease [11]. In addition to its numerous health benefits in humans, linoleic acid plays a critical role in jujube fruits, as it has been shown to enhance the ability of jujube fruit to adapt to low-temperature storage and promote the post-harvest synthesis of aroma compounds, which can have positive effects on overall fruit quality [12,13].

The main enzyme responsible for the synthesis of linoleic acid from oleic acid is microsomal oleoyl phosphatidylcholine desaturase (FAD2), which introduces a double bond at the Δ12 position of oleic acid on phosphatidylcholine and forms linoleic acid on the endoplasmic reticulum (ER). Hence, FAD2 is also referred to as Δ12-desaturaseand and is widely distributed across various plant tissues and organs, which highlights its key role in plant growth and development [14]. Variants of the FAD2 enzyme also have diverse functions in fatty acid modification, including the catalysis of hydroxylation [15] and epoxidation [16] as well as the formation of acetylenic bonds and conjugated double bonds [17,18,19,20]. Some functionally divergent FAD2 enzymes are multi-functional, such as the bifunctional hydroxylase/desaturase from *Lesquerella fendleri* [21] and the tri-functional acetylenase from *Crepis alpina* [22], which can also catalyze the formation of *trans* and *cis* double bonds at the Δ12 position of oleic acid. Although FAD2 has many functions, its catalysis of linoleic acid production is considered its most important function; FAD2 has thus been extensively studied. Since the successful cloning of the *FAD2* gene in *Arabidopsis thaliana* [23], homologous DNA sequences have been isolated and identified in various plants, including soybean (*Glycine max*), peanut (*Arachis hypogaea*), olive (*Olea europaea*), sunflower (*Helianthus annuus*), safflower (*Carthamus tinctorius*), and others. Most plant *FAD2* genes exist as multiple-copy gene families, but only one *FAD2* gene has been identified in Arabidopsis; the greatest number of *FAD2* gene family members (26) has been reported in *Artemisia sphaerocephala* [24,25,26,27,28]. However, few studies have examined *FAD2* genes in jujube, and information on the *FAD2* gene family members in jujube as well as their functions is scarce.

Linoleic acid is one of the key active components contributing to the nutritional and health benefits of jujube, and understanding the molecular mechanism of linoleic acid synthesis can provide insights that can aid the quality and utilization of jujube fruit. Here, we explored the spatial and temporal distribution of fatty acids in jujube fruit and elucidated the molecular mechanism underlying linoleic acid synthesis in wild-type (*Z. jujuba* var. *spinosa* ‘*Tiansuanzao*’) and cultivated (*Z. jujuba* cv. ‘*Xiangfenyuanzao*’) jujube varieties. These varieties were selected for their high kernel rate and differences in fruit size, sensory attributes, and ripening times with the aim of conducting a comprehensive analysis of the fatty acid metabolic profiles in jujube fruit. Bioinformatic and genomic studies, as well as studies of gene expression patterns, revealed the *FAD2* gene family members that govern linoleic acid accumulation. Further study of the function of these gene members was carried out through transient overexpression and virus-induced gene silencing (VIGS) assays in tobacco and jujube fruits. These experimental results enhanced our understanding of the mechanisms underlying linoleic acid synthesis in jujube fruit and will aid future relevant studies.

## 2. Results

### 2.1. Metabolic Profiling of Fatty Acids in Jujube Fruit

The components and content of fatty acids in ‘*Tiansuanzao*’ (TSZ) and ‘*Xiangfenyuanzao*’ (XFYZ) at six developmental stages were detected using gas chromatography-mass spectrometry (GC-MS). A total of 15 main fatty acid components were identified, with carbon chain lengths ranging from 12 carbon atoms to 22 carbon atoms (Tables S1 and S2). Among these components, 12 were detected in the pulp, and 14 were detected in the seeds. The jujube pulp contained the following fatty acids: lauric (C12:0), myristic (C14:0), myristoleic (C14:1), palmitic (C16:0), palmitoleic (C16:1), stearic (C18:0), elaidic (C18:1n9t), oleic (C18:1n9c), linoleic (C18:2n6c), α-linolenic (C18:3n3), arachidic (C20:0), and behenic (C22:0) acid. The seeds lacked myristoleic (C14:1) acid but contained γ-linolenic (C18:3n6), linolenic (C18:3), 11-eicosenoic (C20:1), and *cis*-11,14-eicosadienoic (C20:2) acid.

**Figure 1:**
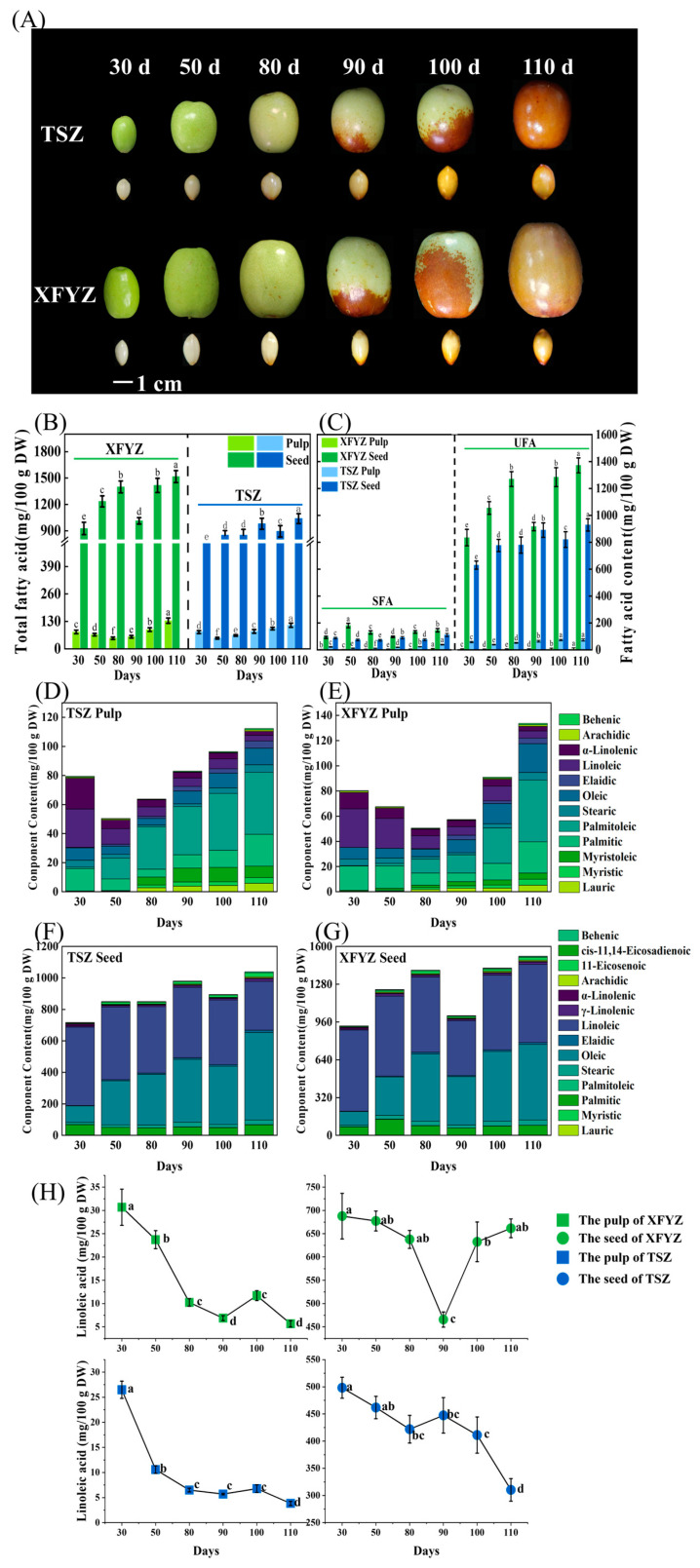
Morphological characteristics of jujube fruits and seeds at different developmental stages and the spatiotemporal distribution of fatty acid components. (**A**) Morphological characteristics of jujube pulp at different developmental stages. (**B**) Total fatty acid content in the pulp and seeds of XFYZ and TSZ at six different stages. (**C**) Content of saturated fatty acids (SFAs) and unsaturated fatty acids (UFAs). (**D**) Content of each component in TSZ pulp during the six stages. (**E**) Content of each component in XFYZ pulp during the six stages. (**F**) Content of each component in TSZ seeds during the six stages. (**G**) Content of each component in XFYZ seeds during the six stages. (**H**) Linoleic acid content in the pulp and seeds during different ripening stages of XFYZ and TSZ. TSZ: ‘*Tiansunzao*’, XFYZ: ‘*Xiangfenyuanzao*’, 30 d, 50 d, 80 d, 90 d, 100 d, and 110 d indicate days after first blooming. Values labeled with different letters indicate significant differences at *p* < 0.05.

The total fatty acid content within the pulp and seeds of XFYZ and TSZ exhibited an increase throughout development, achieving its peak levels on the 110th day following flowering (Figure 1B). A marked disparity was observed between the concentrations of fatty acids in the pulp and the seeds. At maturity, fatty acid levels in the pulp ranged from 112.35 to 133.72 mg/100 g DW, whereas those in the seeds ranged from 1039.16 to 1516.94 mg/100 g DW, indicating that the fatty acid concentrations were ten times higher in the seeds than in the pulp. Little variation was observed in the fatty acid content in the pulp between cultivated XFYZ and wild-type TSZ. However, the fatty acid content in the seeds of XFYZ was higher than that in the wild-type TSZ. Unsaturated fatty acids (UFAs) were more abundant than saturated fatty acids (SFAs) in the pulp and seeds (Figure 1C; Tables S1 and S2). Upon maturation of TSZ, the UFA concentration within the pulp was quantified at 73.50 mg/100 g DW, approximately double that of the SFAs, which registered 38.85 mg/100 g DW. A congruent pattern was discerned in XFYZ, where UFA concentrations stood at 90.55 mg/100 g DW, contrasting with SFA concentrations of 43.17 mg/100 g DW.

The composition of fatty acids in the pulp and seeds is shown in Table S1 and Table S2, respectively. The types of fatty acid components in the seeds were more diverse, but the contents of the main components were more concentrated, focused primarily on two components, oleic acid and linoleic acid (Figure 1F,G). Thirty days after flowering, linoleic acid comprised over 50% of the total content of fatty acids in seeds. However, the distribution of fatty acid components in the pulp was entirely different (Figure 1D,E). The distribution of components in the pulp was more dispersed. Aside from the primary components, palmitic acid, and palmitoleic acid, other components such as oleic acid, linoleic acid, and α-linolenic acid were detected at various stages.

Changes in the concentration of linoleic acid, which was a predominant component of the UFA component in both the pulp and seeds, were similar during the maturation of the XFYZ and TSZ varieties (Figure 1H). From 30 to 80 days after flowering, the content of linoleic acid in the pulp consistently declined; this was followed by slight fluctuations between 90 to 110 days, and it eventually stabilized at levels ranging from 3.84 to 5.66 mg/100 g DW. In the seeds, the concentration of linoleic acid generally decreased as the fruit matured, but the changes were subtle, maintaining a consistently elevated level throughout.

Moreover, fatty acids such as lauric, myristic, myristoleic, stearic, elaidic, arachidic, and behenic acid were detectable in the pulp but remained at concentrations below 10 mg/100 g DW throughout the maturation process (Tables S1 and S2). Despite seeds containing total fatty acid levels approximately ten times higher than the pulp, this significant increase was largely attributed to dominant components. Concentrations of lauric, myristic, palmitoleic, and *cis*-11,14-eicosadienoic in the seeds consistently remained below 2 mg/100 g DW.

### 2.2. Characterization of FAD2 Gene Family Members in Jujube

A BLAST search of the *AtFAD2* gene against the jujube genome revealed a total of five *ZjFAD2* gene members (Table 1). These genes were named *ZjFAD2-1*, *ZjFAD2-5*, *ZjFAD2-8*, *ZjFAD2-9*, and *ZjFAD2-11* based on their chromosomal locations. The coding regions of these genes ranged from 1095 to 1377 bp, and they encoded proteins ranging from 364 to 458 amino acids in length. The molecular weight (Mw) of the encoded proteins varied from 41.14 to 52.00 kDa, and their predicted isoelectric point (pI) ranged from 8.46 to 9.21. The protein encoded by *ZjFAD2-9* had the largest Mw, and that encoded by *ZjFAD2-5* had the lowest pI. Subcellular localization prediction showed that ZjFAD2-8 and ZjFAD2-11 were localized to the nucleus, ZjFAD2-5 and ZjFAD2-9 were located to the endoplasmic reticulum (ER), and ZjFAD2-1 was localized to the plasma membrane.

### 2.3. Chromosomal Mapping and Evolutionary Analysis of ZjFAD2 Gene Family Members

**Figure 2:**
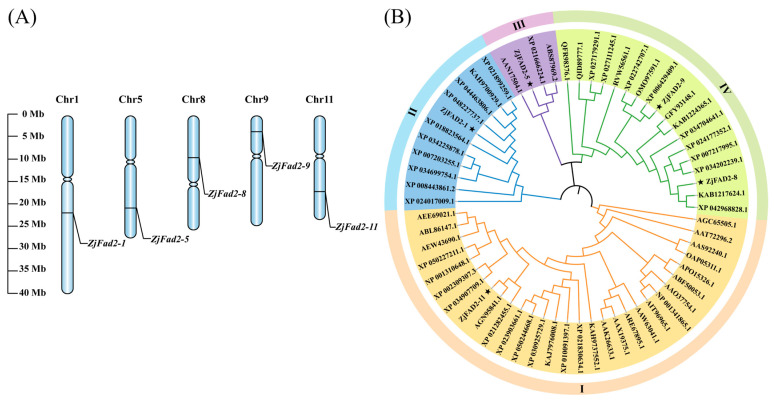
Chromosome localization and phylogenetic analysis of *ZjFAD2* genes. (**A**) Chromosome distribution of the *ZjFAD2* gene family. Chromosome numbers are shown at the top of the chromosomes. *ZjFAD2* genes are labeled at the right of each chromosome. Scale bars on the left indicate the chromosome lengths (Mb). (**B**) Phylogenetic analysis of *ZjFAD2* genes. The phylogenetic tree was generated using MEGA-X software with default parameters using the maximum likelihood (ML) method, and the number of bootstrap replicates was set to 1000. The whole family can be divided into four groups. Jujube genes are indicated by black stars, and the accession numbers of genes from the other species are shown in Table S3.

Analysis of the distribution of *ZjFAD2* genes on chromosomes revealed that five genes were distributed on five different chromosomes and named *ZjFAD2-1*, *ZjFAD2-5*, *ZjFAD2-8*, *ZjFAD2-9*, and *ZjFAD2-11* according to their localization (Figure 2A).

To investigate the phylogenetic relationships among *ZjFAD2* gene family members in plants, we compared the amino acid sequences of 63 FAD2 proteins from 53 plant species and constructed an evolutionary tree (Figure 2B). The 63 FAD2 proteins were categorized into four subgroups based on their evolutionary relationships. Subgroup III and subgroup IV were located within the same evolutionary branch, indicating that they were closely related. In *Ziziphus jujube*, five members of the *ZjFAD2* gene family were identified. As shown in Figure 2B, these proteins were distributed in the four subgroups. Subgroup I contained ZjFAD2-11, subgroup II contained ZjFAD2-1, subgroup III only contained ZjFAD2-5, and subgroup IV contained two members, ZjFAD2-8 and ZjFAD2-9. Furthermore, the amino acid sequences of ZjFAD2-9 and ZjFAD2-11 were highly similar to those of proteins from other species. Specifically, ZjFAD2-9 was highly similar to the FAD2 protein of *Citrus sinensis*, and ZjFAD2-11 was similar to the FAD2 protein of *Hiptage benghalensis*. Additionally, subgroup I, which contained ZjFAD2-1, primarily comprised herbs and vines, including *Arabidopsis thaliana*, *Nicotiana tabacum*, *Salvia hispanica*, and others. Conversely, the other subgroups mainly comprised woody trees, which suggests that *ZjFAD2-11* might be the ancestral gene of the jujube *FAD2* gene family.

### 2.4. Bioinformatic Characterization of ZjFAD2 Gene Sequences

**Figure 3:**
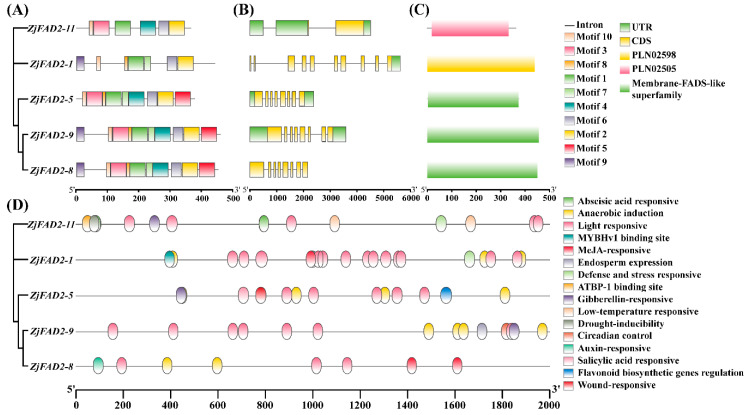
Sequence analysis of *ZjFAD2* gene family members. (**A**) Motif composition and exon/intron structure of *ZjFAD2* genes. (**B**) *ZjFAD2* gene structure. (**C**) Conserved domains of *ZjFAD2* genes. (**D**) Distribution of stress and phytohormone-related *cis*-elements in the putative *ZjFAD2* promoter region. The location of these *cis*-elements was predicted by the PlantCARE database.

The conserved motifs of the *ZjFAD2* gene family were identified by MEME, and a total of 10 conserved motifs were identified among all the analyzed *ZjFAD2*s (Figure 3A). Each gene contained 6–10 motifs, and motifs 1, 2, 6, and 10 were present in all five gene members. The results of the phylogenetic analysis indicate that the genes in the same subgroup had similar conserved motifs. For example, *ZjFAD2-8* and *ZjFAD2-9* belonged to the same subgroup and had highly similar motifs. Among the four *ZjFAD2* genes, *ZjFAD2-5*, *ZjFAD2-8*, and *ZjFAD2-9*, which were all from the same group, had 8 exons, *ZjFAD2-1* had 10 exons, and *ZjFAD2-11* had 2 exons (Figure 3B). The number of introns and exons in *ZjFAD2-11* was significantly lower than that in other family members, and the arrangement of introns and exons was simpler in *ZjFAD2-11* than in other family members, which suggest that *ZjFAD2-11* might be the ancestral gene of the *ZjFAD2* family. This also suggests that this cluster likely evolved via gene replication based on the similarity in exon/intron organization. Further domain analysis using SMART revealed that the *ZjFAD2* family contained only three types of conserved domains, with each member having only one domain (Figure 3C). *ZjFAD2-5*, *ZjFAD2-8*, and *ZjFAD2-9* all had a single membrane-FAD-like superfamily domain, *ZjFAD2-11* contained a single PLN02505 domain, and *ZjFAD2-1* had a PLN02598 domain.

The region 2 kb upstream of the initiation codon (ATG) of the five *ZjFAD2* genes was analyzed online using the PlantCARE database. The promoters of the five *ZjFAD2*s included 16 *cis*-elements (Figure 3D). These *cis*-elements comprised various plant hormone response elements for hormones such as gibberellin, abscisic acid, salicylic acid, auxin, and methyl jasmonate, as well as stress response elements for stress such as hypothermia, herbivory, and other types of stress. The results indicated that *ZjFAD2* genes were involved in light responses and the regulation of growth metabolism, and their expression was regulated by various hormones.

### 2.5. ZjFAD2s Expression Patterns during Jujube Fruit Ripening

**Figure 4:**
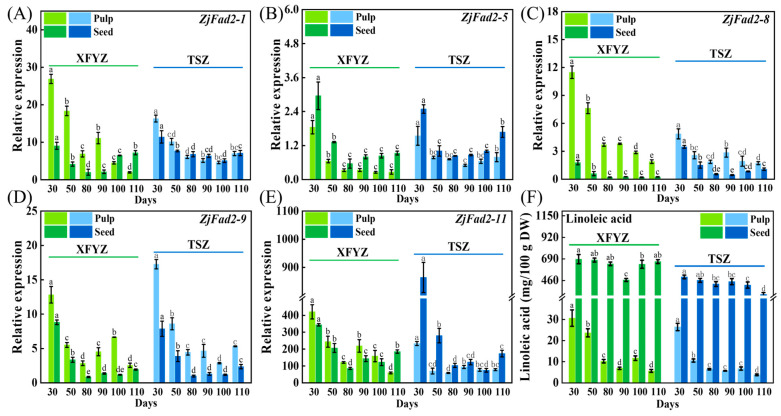
Expression patterns of *ZjFAD2*s and the linoleic acid content in the pulp and seeds during different ripening stages of XFYZ and TSZ. (**A**) Expression pattern of *ZjFAD2-1*. (**B**) Expression pattern of *ZjFAD2-5.* (**C**) Expression pattern of *ZjFAD2-8*. (**D**) Expression pattern of *ZjFAD2-9.* (**E**) Expression pattern of *ZjFAD2-9*. (**F**) Linoleic acid content in the pulp and seeds during different ripening stages of XFYZ and TSZ. TSZ: ‘*Tiansunzao*’, XFYZ: ‘*Xiangfenyuanzao*’, 30 d, 50 d, 80 d, 90 d, 100 d, and 110 d indicate days after first blooming. Data represent the means ± SE of three biological samples. Values labeled with different letters indicate significant differences at *p* < 0.05.

To clarify the roles of *ZjFAD2* genes in linoleic acid biosynthesis, the expression of these five genes was analyzed in both the pulp and seeds at various stages of maturity (Figure 4). The relative expression of *ZjFAD2-11* was notably higher that of the other four genes (Figure 4E). Conversely, the relative expression of *ZjFAD2-5* was the lowest (Figure 4B).

During the entire developmental period, the expression levels of all five genes were highest at 30 days after flowering. This was followed by a substantial decline from 30 to 80 days after flowering and subsequently varied divergently between 90 and 110 days. This was consistent with the observed variation in the linoleic acid content (Figure 4F). Specifically, the expression of most genes in the pulp decreased from 90 to 110 days after flowering, such as *ZjFAD2-1*, *ZjFAD2-8*, and *ZjFAD2-11* (Figure 4A,C,E). In the seeds, the expression of most genes slightly increased during this period, such as *ZjFAD2-1*, *ZjFAD2-5*, and *ZjFAD2-11* (Figure 4A,B,E). Furthermore, comparing the relative expression of five genes in different tissues of two varieties, in XFYZ, the genes *ZjFAD2-1*, *ZjFAD2-8*, *ZjFAD2-9*, and *ZjFAD2-11* exhibited higher expression levels in the pulp compared to seeds, while *ZjFAD2-5* manifested higher expression in seeds. In TSZ, *ZjFAD2-1*, *ZjFAD2-8*, and *ZjFAD2-9* were more pronounced in the pulp, and *ZjFAD2-5* and *ZjFAD2-11* were dominantly expressed in seeds. These findings suggest that the regulatory mechanisms of these genes differ in the pulp and seeds of jujube fruit.

**Figure 5:**
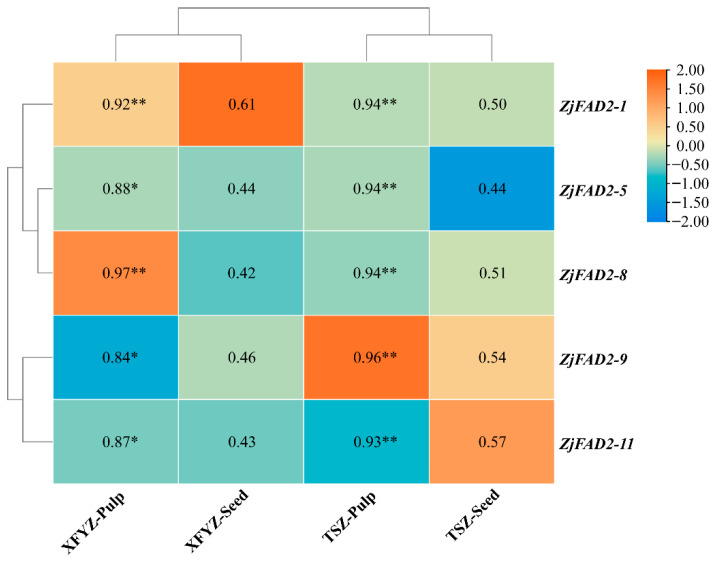
Heat map of the correlation between the relative expression of the five *ZjFAD2* genes and the linoleic acid content in the pulp and seeds. TSZ: ‘*Tiansunzao*’ XFYZ: ‘*Xiangfenyuanzao*’. Data were analyzed using Student’s *t*-test: * *p* < 0.05, ** *p* < 0.01.

Upon contrasting the expression patterns of the five genes with the spatiotemporal distribution of linoleic acid content, it was discerned that the relative expression trends of these five *ZjFAD2* genes corresponded harmoniously with the fluctuations in linoleic acid levels (Figure 5). A deeper correlation analysis subsequently revealed a significant positive association between these genes and linoleic acid synthesis. Intriguingly, the correlation appeared more robust in the pulp than in the seeds. This observation lends support to the notion that the *ZjFAD2* gene family members potentially played a role in enhancing the linoleic acid accumulation in jujube while also suggesting distinct regulatory roles of *ZjFAD2* in linoleic acid synthesis in seeds versus pulp.

### 2.6. Functional Verification of the ZjFAD2 Genes in Plants

To validate the in vivo function of the five *ZjFAD2*s, heterologous transient expression assays were carried out in tobacco, and homologous transient expression assays were conducted in jujube fruits.

**Figure 6:**
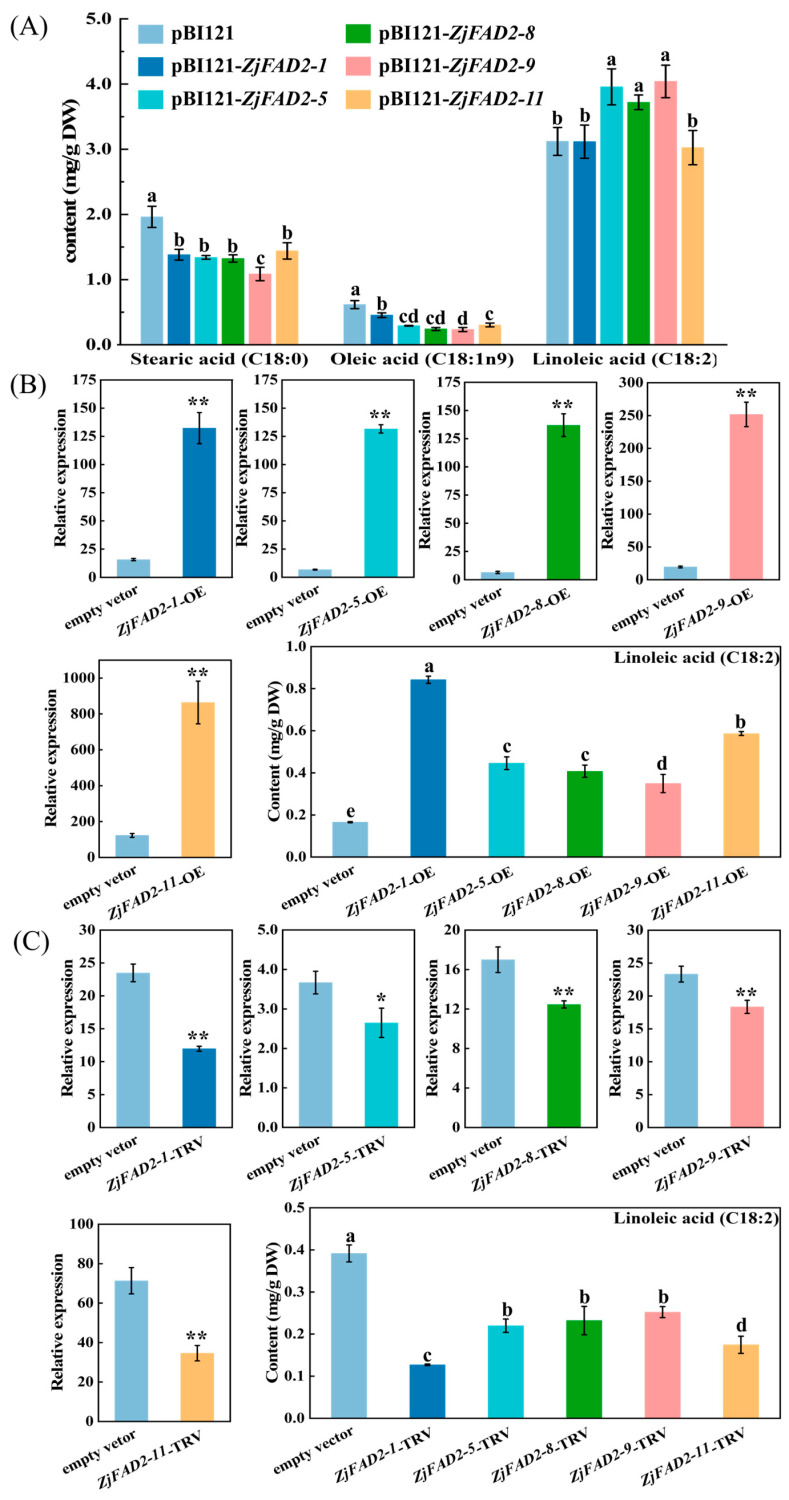
*ZjFAD2* genes participate in linoleic acid biosynthesis in ‘*Dongzao*’. (**A**) Changes in the levels of substances related to the linoleic acid synthesis pathway after the overexpression of five *ZjFAD2* genes in tobacco. (**B**) Overexpression of *ZjFAD2* genes in jujube fruits. (**C**) VIGS of *ZjFAD2* genes in jujube fruits. The values of stearic, oleic, and linoleic acid are the means ± SE of three replicates. Values labeled with different letters indicate significant differences at *p* < 0.05. Data were analyzed using Student’s *t*-test: * *p* < 0.05, ** *p* < 0.01. The values shown are the means ± SE, as assessed by qRT-PCR of three replicates.

As shown in Figure 6A, the experimental results revealed a significant increase in the content of linoleic acid in tobacco leaves overexpressing *ZjFAD2-5*, *ZjFAD2-8*, and *ZjFAD2-9* relative to the control group (increases of 26.8%, 19.2%, and 29.5%, respectively). However, no significant changes were observed in the remaining two genes. Furthermore, comparison of the levels of stearic acid and oleic acid, which act as upstream substrates in the fatty acid synthesis pathway involving *FAD2* genes, revealed that the overexpression of all five genes led to a decrease in the content of stearic acid and oleic acid. The transient overexpression of *ZjFAD2-9* resulted in the most substantial increase in the linoleic acid content and the greatest reduction in stearic acid and oleic acid levels, suggesting that it plays a key role in linoleic acid synthesis.

In jujube fruits, transient overexpression experiments revealed a noteworthy upregulation in the relative expression of all five genes compared with the empty vector control. Additionally, the content of linoleic acid was significantly higher in all overexpressed samples than in the control. The linoleic acid content in *ZjFAD2-1* and *ZjFAD2-11* was two to three times higher in the overexpressed samples than in the control (Figure 6B). Similarly, after VIGS, the expression levels of all five genes and the linoleic acid content decreased (Figure 6C). These results indicate that all five *ZjFAD2* genes promote linoleic acid synthesis and play essential roles in this process.

## 3. Discussion

### 3.1. Fatty Acid Metabolite Profiling in Jujube Fruit

The various bioactive components found in jujube provide key evidence for its health benefits [29]. Jujube fatty oil, especially the fatty oil in jujube seeds, has received increased attention for its active ingredients with sedative, hypnotic, and anti-inflammatory effects. A total of 33 fatty acid components have been identified in jujube pulp, with carbon chain lengths ranging from 7 to 28 carbon atoms [30]. However, few studies have examined the metabolic mechanisms and biosynthetic pathways of fatty acids in jujube.

Qualitative and quantitative analyses of several major fatty acids in TSZ and XFYZ were conducted in different developmental stages and tissues (Tables S1 and S2). Previous studies have shown that palmitic acid and oleic acid are the main fatty acids in jujube pulp, which is consistent with the results of our study [31]. The results of our study show that jujube seeds contain a greater variety of components and a higher fatty acid content, especially linoleic acid and oleic acid, which possess higher nutritional and medicinal value [32]. However, the fatty acid content in jujube seeds observed in our study was notably lower than that observed in previous studies [33]. This discrepancy might be attributed to the use of tender and fresh seeds in our experiments, whereas dried jujube varieties have been used in previous studies. As the fruit gradually matures, the content of oleic acid increases, and the content of linoleic acid decreases, indicating that the conversion from oleic acid to linoleic acid mainly occurs during the early stages of fruit development; such information provides insights that will aid further study of the mechanism of linoleic acid synthesis. The above results revealed the spatial metabolic characteristics of fatty acids in jujube and provide a basis for exploring the metabolic mechanisms of fatty acid compounds in jujube. These findings are consistent with the results of previous studies aimed at identifying fatty acid compounds in jujube.

### 3.2. Genome-Wide Identification and Characterization of ZjFAD2 Genes in Jujube

In plants, *FAD2* gene families are ubiquitous and play a critical role in the synthesis of unsaturated fatty acids. In the model plant *Arabidopsis thaliana*, *AtFAD2* was the first identified *FAD2* gene, and it is located on a simplex chromosome [23]. Few studies of *FAD2* family members in jujube have been conducted; only a few transcriptome analyses have mentioned the *FAD2* gene in the fatty acid synthesis pathway. In this study, physicochemical property analysis, subcellular localization prediction, and chromosome localization analysis were conducted on the five *FAD2* members identified in jujube, and this provided basic information on these genes. Notably, among the five members, *ZjFAD2-5* and *ZjFAD2-9* were predicted to be located in the ER, which is consistent with the functional site of the FAD2 enzyme. However, the prediction results have certain limitations. Different prediction platforms might produce varying outcomes, and the specific subcellular localization should be experimentally confirmed. Phylogenetic analysis indicated that these five genes can be divided into four subgroups, and ZjFAD2-11, which is closely related to FAD2 proteins from herbaceous plants, might be the ancestral gene of this family. Additionally, analysis of gene structure, conserved protein domains, and *cis*-regulatory elements suggests that genes within the same subgroup had similar structures and conserved protein domains, which is consistent with the findings of previous research. Analysis of the upstream promoter regions reveals that these five genes respond to various environmental signals, indicating that they potentially play a role in plant stress resistance. This observation will aid future studies of the functions of these genes.

### 3.3. The Association between ZjFAD2 Gene Expression Patterns and Linoleic Acid Accumulation

Previous research has indicated that the *FAD2* gene family has undergone gene duplication, gene inactivation, and other events, which has resulted in differentiation in structure and expression patterns among gene members [34]. Other studies have noted that the expression of *FAD2* genes can be classified as constitutive and seed-specific, although the expression patterns and functions of *FAD2* genes in plants vary temporally and spatially [35]. The findings of this study revealed that the expression of *ZjFAD-5* was higher in the seeds of both varieties compared with the pulp, and the expression of *ZjFAD2-8* was significantly higher in the pulp than in the seeds, highlighting the temporal and spatial variation in the expression of *FAD2* genes.

In peanuts, the expression of *AhFAD2* is downregulated during ripening, and the decrease in the linoleic acid content is correlated with the expression of *FAD2* [36]. Similarly, in *Olea europoea*, a comparison of the linoleic acid content in different varieties revealed that *OeFAD2* expression is upregulated in high-linoleic-acid varieties [26]. These results strongly indicate that the role of *FAD2* is a key gene in the synthesis of linoleic acid in various plant species, suggesting that the role of the *FAD2* gene in linoleic acid biosynthesis is functionally conserved. Correlation analysis indicated that there is a positive relationship between gene expression and the content of linoleic acid, suggesting that the expression of *ZjFAD2* genes could enhance the linoleic acid content (Figure 5). However, the biosynthesis of linoleic acid is a complex process. The regulation of gene expression is the initial step in the synthesis process, and the expression of genes can also be affected by post-transcriptional modifications, such as protein methylation and deacetylation. This is a limitation of our study that merits consideration.

### 3.4. The Mechanistic Roles of ZjFAD2 Genes in Linoleic Acid Biosynthesis

The *FAD2* gene plays a role in various processes, including fatty acid synthesis, growth and development, and stress resistance. However, the most important role of FAD2 involves catalyzing the conversion of oleic acid into linoleic acid. The function of this target gene has been successfully validated in diverse plants, including walnut, peanut, soybean, safflower, and Arabidopsis.

In this study, five *FAD2* genes from jujube were tested in tobacco and jujube fruit. The results showed that these genes promote linoleic acid synthesis, which is consistent with the results of previous studies of rapeseed and soybean. Several previous studies have shown that the heterologous expression of *FAD2* genes from these plant sources can increase the linoleic acid content in tobacco to varying degrees. Overexpressing *ZjFAD2-5*, *ZjFAD2-8*, and *ZjFAD2-9* in tobacco leaves notably increased linoleic acid more than the other two. Conversely, overexpressing *ZjFAD2-1* and *ZjFAD2-11* in jujube fruit resulted in a greater linoleic acid increase than the other three *FAD2* genes. This difference might be attributed to the distinct expression patterns and regulatory mechanisms of *FAD2* genes in the two plant species. These findings are consistent with the results of previous studies. For example, after transforming the *AsFAD2-4* and *AsFAD2-13* genes into Arabidopsis *FAD2* mutants in safflower, no oleic acid desaturase activity was detected, but the expression of *AsFAD2-13* was high in various tissues of safflower [37]. Therefore, some *FAD2* genes might only be expressed in specific tissues or under particular conditions, and their specific functions require further investigation. However, interpretation of the results of transient expression experiments requires caution given that their ability to reflect the full physiological functions of these genes is limited due to restrictions in temporal and spatial expression patterns. Additionally, in the tobacco transient expression system, the expression of *ZjFAD2* genes led to a decrease in the content of upstream substrates (stearic acid and oleic acid), indicating negative feedback inhibition, which is consistent with the results of previous studies.

In sum, the transient expression of *ZjFAD2* genes provided insights into linoleic acid biosynthesis in jujube fruit. Stable genetic transformation is required to clarify the functions of these genes. Although our study was focused on linoleic acid synthesis in fruit, the entire jujube plant, including roots, stems, and leaves, contributes to linoleic acid accumulation; thus, studies of the roles of various plant parts in linoleic acid accumulation are needed. Especially, given that we detected a fatty acid content in the seeds that was ten times greater than in the pulp, studying the functions of these five genes in the seeds becomes even more urgent and necessary.

## 4. Materials and Methods

### 4.1. Plant Materials

The jujube fruits TSZ and XFYZ used in the experiment were collected from the Jujube Experiment Station of Northwest A&F University in Qingjian County, Shaanxi Province (37.13° N, 110.09° E), China. Pulp and seed samples were collected at six stages of development: days 30, 50, 80, 90, 100, and 110 after the start of flowering. Fifteen randomly selected fruits at each stage comprised one replicate, and three replicates were collected. The collected materials were immediately frozen in liquid nitrogen and stored in a refrigerator at −80 °C.

### 4.2. Extraction and Determination of Fatty Acids by GC-MS

The composition of the medium and long-chain fatty acids in the pulp and seeds was determined following the method described by Wu et al. [38]. The Folch method [39] was used to extract fatty acids from the samples. Subsamples of 0.2 g from each sample were used for the fatty acid extraction, and three biological replicates were performed. To extract the fatty acids, 4 mL of 1% sulfuric acid–methanol was added to the extracted sample. The mixture was thoroughly shaken and heated in a water bath at 80 °C for approximately 30 min until the oil was completely dissolved. Subsequently, 2 mL of *n*-hexane was added, and distilled water was used to collect the supernatant. Using methyl tridecanoate as a standard, an ISQ gas chromatography/mass spectrometer (Thermo Fisher Scientific Inc., Waltham, MA, USA) was used to determine the composition and content of fatty acids. The determination of total fatty acids was performed using a TRACE1310-ISQLT system with an HP-INNOWAX column (30 m × 0.25 m × 0.25 μm) as described by Fei et al. [40].

The qualitative analysis of fatty acids was conducted by comparing their mass spectral fragmentation with the National Institute of Standards and Technology and the retention times of authentic methyl ester mixture standards (C4-C24, Shanghai yuanye Bio-Technology Co., Ltd., Shanghai, China). or quantification, both the external standard method and the internal standard method based on peak area were used. The content of each fatty acid was expressed as mg per 100 g of dry sample (mg/100 g DW).

### 4.3. Identification and Characterization of ZjFAD2 Genes

To identify the *FAD2* genes in jujube, two FAD2 protein sequences of *Arabidopsis thaliana* were downloaded from the Swissprot database online: https://www.uniprot.org (accessed on 13 May 2023) and used as queries for a local BLAST analysis. The jujube genome dataset was obtained from our previous study [41]. The BLAST output results were further aligned by protein BLAST in NCBI online: https://blast.ncbi.nlm.nih.gov (accessed on 13 May 2023), and the FAD2 domains were confirmed using the Batch Web CD-Search Tool online: https://www.ncbi.nlm.nih.gov/Structure/bwrpsb/bwrpsb.cgi (accessed on 13 May 2023). Next, the Mw and pI of these validated proteins were predicted using Expasy online: http://web.expasy.org/protparam/ (accessed on 10 May 2023) [42]. We used the WOLF PSORT website: https://wolfpsort.hgc.jp/ (accessed on 11 May 2023) to predict the subcellular localization of *ZjFAD2* genes [43], and the chromosomal localization of these genes was analyzed and visualized using TBtools-II v2.003 software [44].

### 4.4. Phylogenetic Analysis of Proteins

Based on the two publicly available AtFAD2 protein sequences, we conducted a comparison and screening of protein sequences from different species. These sequences were obtained from the EnsemblPlants online: https://plants.ensembl.org/index.html (accessed on 18 May 2023) and Phytozome online: https://phytozome-next.jgi.doe.gov/ (accessed on 18 May 2023) databases [45], using TBtools-II v2.003 software along with the NCBI website. A total of 63 FAD2 proteins from 53 species, including herbaceous plants (*Arabidopsis thaliana*, *Nicotiana tabacum*, *Carthamus tinctorius*, and *Arachis hypogaea*), trees (*Triadica sebifera*, *Vernicia fordii*, and *Populus alb*), and fruit trees (*Prunus avium*, *Citrus sinensis*, *Prunus persica*, and *Morella rubra*) were obtained after screening. These proteins were used for sequence alignment and the construction of a protein phylogenetic tree in MEGA-X software. The species and protein information are shown in Table S3. The maximum likelihood method (1000 bootstrap replicates) was used, and the best-fit model was LG + G. The evolutionary tree was visualized using TBtools-II v2.003 software and iTOL online: https://itol.embl.de/ (accessed on 28 May 2023) [46].

### 4.5. Gene Structure and Protein Structure Analysis

We conducted gene structure analysis of *ZjFAD2* genes using TBtools-II v2.003 and analyzed motifs in ZjFAD2 family proteins using MEME online: http://meme-suite.org/tools/meme (accessed on 18 May 2023); information on the conserved domains of *ZjFAD2* genes was obtained from the NCBI database and further domain analysis using SMART online: http://smart.embl-heidelberg.de/ (accessed on 18 May 2023). The promoter sequence 2000 bp upstream of the gene translation initiation site was extracted and submitted to the PlantCARE database online: http://bioinformatics.psb.ugent.be/webtools/plantcare/html/ (accessed on 19 May 2023) [47] for *cis*-element analysis, and TBtools-II v2.003 was used to make a statistical map of the *cis*-elements.

### 4.6. Expression Analysis of ZjFAD2 Genes during the Ripening of Jujube

The TSZ and XFYZ fruits and seeds were harvested at six ripening stages for qRT-PCR analysis to characterize gene expression. Total RNA was extracted from the samples and used as the template for first-strand cDNA synthesis in the qRT-PCR. Primer 5.0 software was used to design primers (Table S4). *ZjUBQ1* and *ZjUBQ2* were the internal standards [48]. qRT-PCR was conducted on the Bio-Rad IQ5 instrument using a SYBR Premix Ex Taq Kit (TaKaRa). All reactions were performed using the following thermal cycling conditions: 95 °C for 3 min, followed by 35 cycles of 95 °C for 5 s and 56 °C for 30 s. Gene expression levels were analyzed using the 2^−ΔΔCT^ method. All results were calculated based on three biological replicates.

### 4.7. Transient Overexpression or Silencing of ZjFAD2 Genes in Jujube Fruit and Nicotiana tabacum Leaves

Transient functional verification was carried out following the method described in a previous study [2]. The full complementary DNA of five *ZjFAD2* genes was inserted into the multiple cloning site (*Bam*HI-*Sma*I) of the destination vector pBI121. The primers are shown in Tables S5 and S6. The 200 bp fragments of *ZjFAD2*s were selected using the VIGS design tool online: https://vigs.solgenomics.net/ (accessed on 11 April 2023) and inserted into the multiple cloning site (*Sac*I-*Xba*I) of the TRV2 vector. The primers are listed in Table S7. The constructs were then transformed into *Agrobacterium tumefaciens* (GV3101). Positive clones were selected, grown, and then washed and resuspended in infiltration buffer (10 mM MES at pH 5.6, 10 mM MgCl_2_, 100 µM acetosyringone) to an OD_600_ of 0.6 for injection. In addition, VIGS requires the resuspension mixture pTRV2-*ZjFAD2*s and pTRV1 to be mixed at a 1:1 ratio before injection.

‘*Dongzao*’(DZ) jujube fruits, cultivated at the Jujube Experiment Station of Northwest A&F University in Dali County, Shaanxi Province (34.88° N, 110.04° E), China, were injected with the infiltration mix at the white mature stage. The control groups were treated with either the empty vector pBI121 or pTRV2 + pTRV1. Two injection sites were utilized on each fruit, and a total of six biological replicates were conducted. Three weeks after injection, the injected fruits were harvested from the trees for fatty acid determination. ‘NC89’ tobacco at the six-leaf stage was selected for injection, and the pBI121 empty vector was injected as the control group. After injection, all plants were kept away from light for 12 h; they were then cultivated normally in a climate chamber for one week before being collected for fatty acid analysis.

## 5. Conclusions

Jujube is a unique economically important fruit tree in China. Its fruits and seeds contain abundant fatty acids, and linoleic acid is an essential polyunsaturated fatty acid. A series of studies were conducted to elucidate the metabolic patterns and mechanisms underlying the synthesis of linoleic acid in jujube fruit. Spatiotemporal variation in the composition and content of fatty acids in the wild-type TSZ and cultivated variety XFYZ was elucidated. C16 fatty acids are the predominant fatty acids in the pulp, whereas C18 fatty acids are predominant in the seeds; levels of C18 fatty acids were 10 times higher in the seeds than in the pulp. Linoleic acid was the main polyunsaturated fatty acid in jujube, and its content decreased during fruit maturation. We identified five *ZjFAD2* genes (*ZjFAD2-1*, *ZjFAD2-5*, *ZjFAD2-8*, *ZjFAD2-1*, *ZjFAD2-9*, and *ZjFAD2-11*), and they were divided into four subgroups based on phylogenetic analysis. Subsequently, qRT-PCR analysis of the five genes showed that the expression of *ZjFAD2* was highly correlated with linoleic acid synthesis. Moreover, both heterologous and homologous transient expression analyses indicated that the five *ZjFAD2*s positively regulated linoleic acid synthesis in jujube fruit to varying degrees. *ZjFAD2-11* and *ZjFAD2-1*, which exhibited higher relative expression and more significant regulatory effects compared with the other genes, were speculated to be the key genes in the *ZjFAD2* gene family. These studies have enhanced our understanding of fatty acid synthesis in jujube and provided insights into the molecular mechanism of linoleic acid synthesis in jujube; the results of our study have implications for improving the fruit quality of jujube fruit via breeding programs.

## Figures and Tables

**Table 1:** Basic characteristics of *ZjFAD2* genes.

Gene Name	Coding Regions/bp	Isoelectric Point	Molecular Weight/kDa	Amino Acid/aa	Subcellular Location Predicted
*ZjFAD2-1*	1326	9.21	51.36	441	Plas
*ZjFAD2-5*	1134	8.46	43.91	377	ER
*ZjFAD2-8*	1359	8.89	51.54	452	Nuc
*ZjFAD2-9*	1377	8.66	52.00	458	ER
*ZjFAD2-11*	1095	8.96	41.14	364	Nuc

Abbreviations are used to indicate the subcellular localization of ZjFAD2 proteins: “Plas” refers to the plasma membrane, “ER” refers to the endoplasmic reticulum, and “Nuc” refers to nuclear.

## Data Availability

Data are contained within this article and the Supplementary Material.

## Supplementary Materials

The following supporting information can be downloaded at: https://www.mdpi.com/article/10.3390/ijms242015479/s1.
